# Nano-ionic Solid Electrolyte FET-Based Reservoir Computing for Efficient Temporal Data Classification and Forecasting

**DOI:** 10.1021/acsami.5c00092

**Published:** 2025-03-07

**Authors:** Ankit Gaurav, Xiaoyao Song, Sanjeev Kumar Manhas, Partha Pratim Roy, Maria Merlyne De Souza

**Affiliations:** † Indian Institute of Technology Roorkee, Roorkee, 247667, India; ‡ University of Sheffield, Sheffield, S13JD, United Kingdom

**Keywords:** physical reservoir computing, solid electrolyte FET, temporal data, classification, forecasting, edge systems

## Abstract

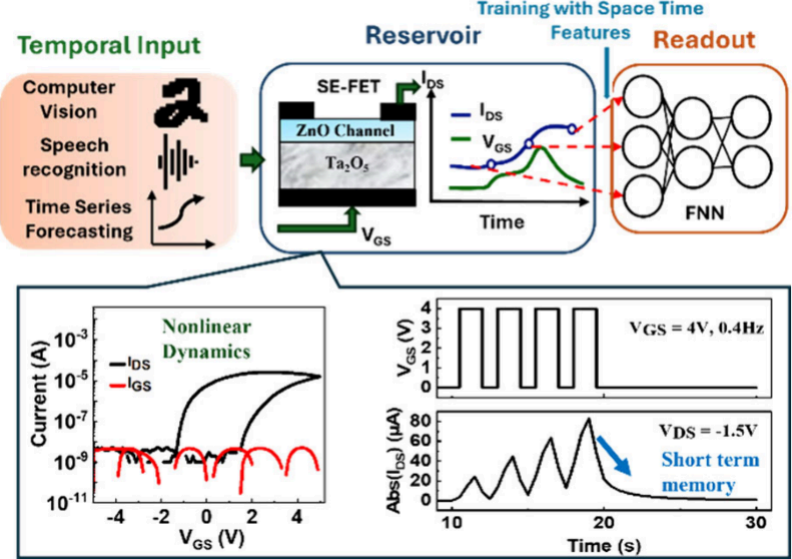

Physical dynamic reservoirs are well-suited for edge systems, as they can efficiently process temporal input at a low training cost by utilizing the short-term memory of the device for in-memory computation. However, the short-term memory of two-terminal memristor-based reservoirs limits the duration of the temporal inputs, resulting in more reservoir outputs per sample for classification. Additionally, forecasting requires multiple devices (20–25) for the prediction of a single time step, and long-term forecasting requires the reintroduction of forecasted data as new input, increasing system complexity and costs. Here, we report an efficient reservoir computing system based on a three-terminal nano-ionic solid electrolyte FET (SE-FET), whose drain current can be regulated via gate and drain voltages to extend the short-term memory, thereby increasing the duration and length of the temporal input. Moreover, the use of a separate control terminal for read and write operation simplifies the design, enhancing reservoir efficiency compared to that in two-terminal devices. Using this approach, we demonstrate a longer mask length or bit sequence, which gives an accuracy of 95.41% for the classification of handwritten digits. Furthermore, this accuracy is achieved using 51% fewer reservoir outputs per image sample, which significantly reduces the hardware and training cost without sacrificing the accuracy of classification. We also demonstrate long-term forecasting by using 50 previous data steps generated by an SE-FET-based reservoir consisting of four devices to predict the next 50 time steps without any feedback loop. This approach results in a low root-mean-square error of 0.06 in the task of chaotic time-series forecasting, which outperforms the standard linear regression machine learning algorithm by 53%.

## 1 Introduction

**Figure 1:**
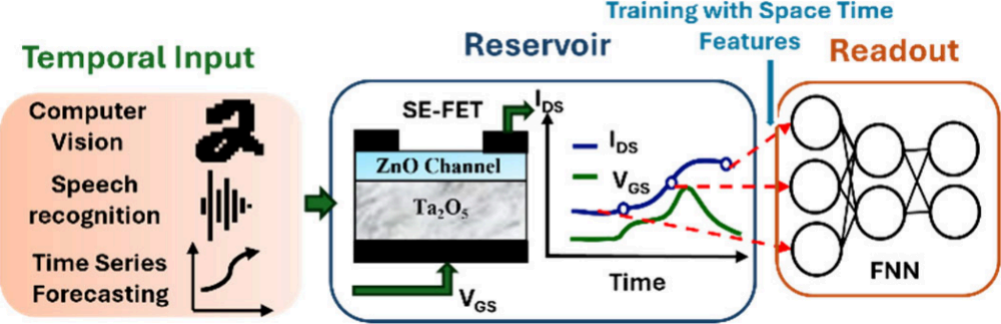
SE-FET reservoir system’s framework demonstrates in-memory computing capabilities for processing temporal input.

Recurrent neural networks (RNNs)^1,2^ excel in handling temporal input compared to traditional feedforward neural networks (FNNs),^3^ but their cyclic connections introduce vanishing and exploding gradients, making the training computationally expensive. To address these challenges, variations of RNNs have been proposed, i.e., long short-term memory^4^ and reservoir computing (RC).^5,6^ A dynamic physical reservoir leverages short-term memory (fading memory) to transform temporal inputs into space–time-dependent features.^7^ The states of the device (i.e., read current (*I*_ds_)) represent the feature space of the reservoir that is used to train the readout network, as shown in Figure 1. This fundamental idea of RC is versatile: one can solve many tasks such as classification of spoken digits,^8−11^ handwritten digits,^12−14^ and chaotic time-series forecasting.^9,15^ However, most of these physical reservoirs exhibit an accuracy of 83–86% when applied to real-world challenges, such as classification of handwritten digits using the MNIST data set.^16^ To improve accuracy, various methods have been demonstrated, i.e. two different pulse rates for the same input sequence,^12^ and a fast and slow read at the end of each sequence (i.e., reading the reservoir states twice).^13^ These methods result in doubling the reservoir output per handwritten digit, which subsequently increases the training costs and complexity of the reservoir for a 2–3% improvement in performance.

In another approach, the readout network consisting of an FNN with one or two hidden layers was used instead of logistic regression^17,18^ to give 95–96% accuracy without increment in the reservoir output per input digit. Most of these reservoirs use a volatile two-terminal memristor device in which a fast diffusive species (e.g., Ag) is used to achieve fading memory to generate reservoir states.^12,13,15,19^ However, in two-terminal memristor reservoirs, the input signal duration and interval are constrained by the time span of memory decay, typically a few milliseconds. This severely limits the ability to handle longer sequences, usually 4-bit for classification of MNIST digits. In another study,^17^ RC was demonstrated using nonvolatile memristors combined with circuit elements such as resistors and capacitors to achieve not only fading memory but also longer time constants, which results in additional cost and complexity. Alternatives to two-terminal memristors are three-terminal devices such as the solid electrolyte FET (SE-FET)^20^ and leaky FinFET.^18^ These devices offer benefits of low power consumption by operating in the off state, i.e., in the absence of a gate pulse.^21^ In an SE-FET, writing in the off-state minimizes power consumption to nanowatt (nW) levels, even with a high *L*/*W* ratio of the transistor. When benchmarked against other ReRAM devices in our previously reported work,^21^ it achieves a competitive 8 nJ per transition, demonstrating lower power consumption than filamentary devices. Additionally, the use of a separate control terminal for read and write operations simplifies the reservoir implementation compared with two-terminal devices. On the other hand, in a leaky FinFET, an absence of tunneling oxide led to reduced retention time, resulting in short-term memory of a few microseconds. In our previous work based on an SE-FET based reservoir,^14^ the classification accuracy was enhanced by reading the reservoir states after each input in a sequence, rather than at the end. Although this approach improved the accuracy to 91.19%, it did not reduce the number of reservoir outputs per handwritten digit.

Apart from temporal data classification, time-series forecasting is also a crucial application of physical reservoir computing, enabling the prediction of future data points based on historical patterns and trends. A previous study^9^ of time-series prediction using two terminal memristors relied on a reservoir consisting of 20 devices, where 50 previous states of each device were used to predict the subsequent time step. Moreover, the predicted data were fed back into the reservoir as new input for autonomous prediction. However, this procedure increases the system complexity, as it requires continuous feedback of predicted data to the reservoir for longer-term prediction. This technique was shown to work for only 60–70 time steps of autonomous prediction, after which the predicted signal diverges from the correct value. To address this limitation, an update stage was introduced: the true input (instead of the predicted value) was used for 25 time steps after 50 steps of autonomous prediction, further increasing system complexity. Similarly, another study using a tin monoxide thin-film transistor (SnO TFT)-based reservoir incorporates a feedback loop and update cycle to make long-term predictions.^22^ In SnO TFT, electron trapping and time-dependent detrapping at the channel interface cause the device to exhibit short-term memory. Further, in another work,^15^ a reservoir consisting of 25 memristor devices with increased hardware costs was reported, where four previous states of each device were used to perform only short-term prediction.

This work demonstrates a highly efficient physical reservoir computing system based on a three-terminal nano-ionic solid electrolyte ZnO/Ta_2_O_5_ field-effect transistor (SE-FET) for temporal data classification and chaotic time-series forecasting. By modulating gate and drain voltages, we significantly extend the memory effect in SE-FET reservoirs, achieving memory decay time (τ > 40 s). This enhancement enables improved processing of longer temporal sequences while maintaining a high classification accuracy (95.41%) and reducing the required reservoir outputs per input by 51%. Furthermore, we experimentally demonstrate, for the first time, the capability of SE-FET-based reservoirs to process real-world analog signals, moving beyond our previous studies limited to binary sequences.^14^ Additionally, we introduce a novel long-term forecasting framework, utilizing data from the past 50 steps across four SE-FET devices to predict the next 50 steps, achieving high predictive accuracy without the need for feedback loops or update cycles. A systematic device-to-device variability analysis further provides insights into interface dynamics and their impact on the performance of the SE-FET, ensuring reliability for large-scale reservoir computing applications. These advancements enhance computational efficiency while maintaining high predictive accuracy, demonstrating the potential of SE-FET-based reservoir computing for real-world analog signal processing and scalable neuromorphic systems, especially suited for longer time scale applications such as biomedical and the Internet of Robotic Things.

## 2 Experimental Methods

### 2.1 Experimental Fabrication and Device Mechanism

**Figure 2:**
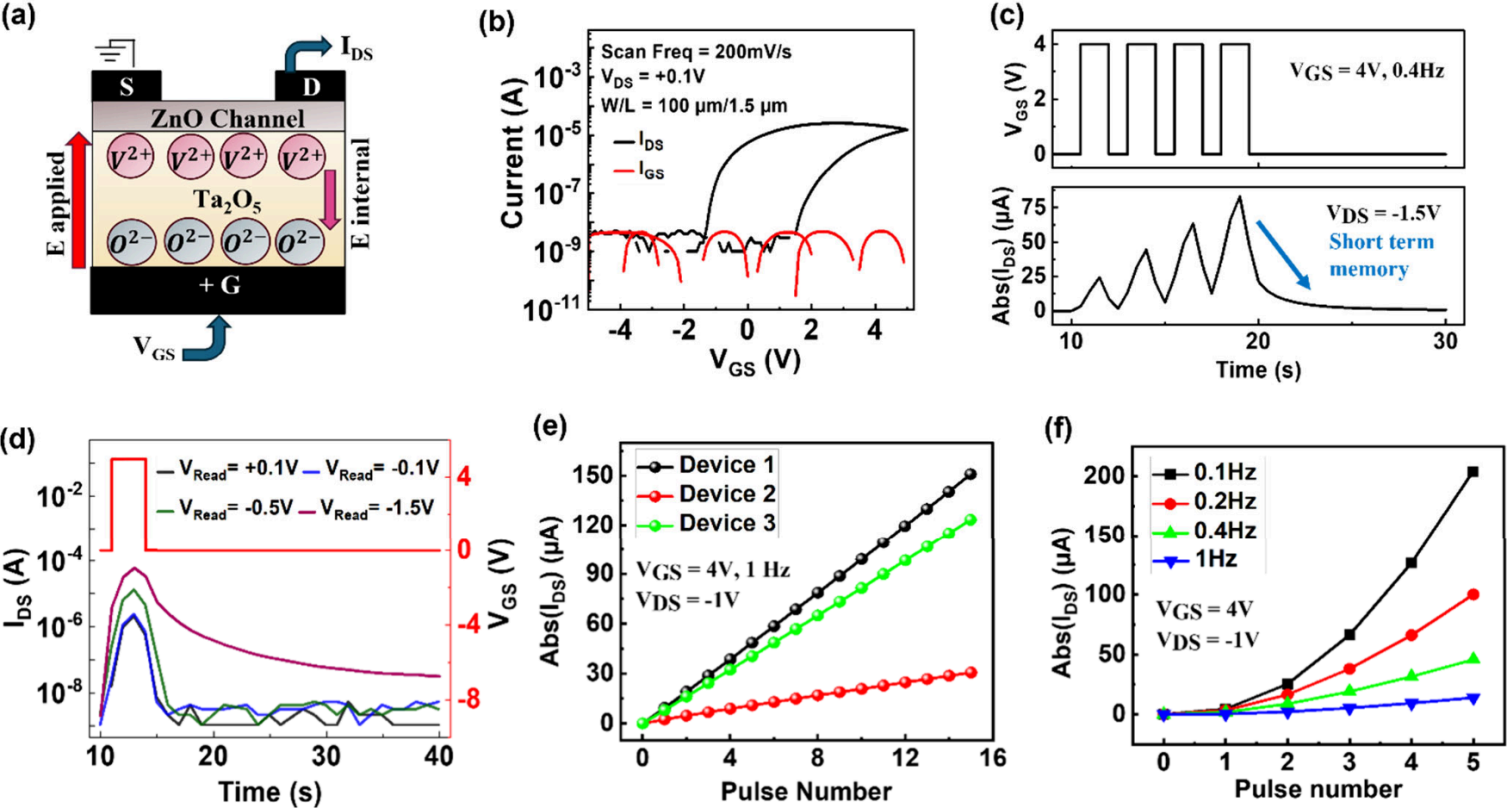
(a) Schematic of the SE-FET, showing vacancies (V^2+^) and oxygen ions (O^2–^) separated at the respective opposite interfaces of Ta_2_O_5_, upon application of a gate voltage. (b) Measured transfer characteristics of the fabricated ZnO/Ta_2_O_5_ SE-FET as a function of scan frequency and the corresponding gate current characteristics. (c) Short-term memory in the SE-FET. The device was first programmed by five write pulses of *V*_GS_ = 4 V at 0.4 Hz, and its response *I*_DS_ is measured by read pulses of *V*_DS_ = −1.5 V. (d) A single write pulse of 5 V is used to program the device, and its subsequent *I*_DS_ is measured using four different *V*_Read_. The results show varying memory decay times (τ), with *V*_Read_ = −1.5 V achieving τ > 40 s. (e) Device-to-device variation across three different SE-FET devices when subjected to the same input of *V*_GS_ = 4 at 1 Hz. (f) Response of the SE-FET when subjected to input at four different frequencies, showing the uniqueness of the output response.

Our bottom-gated SE-FET is fabricated on a glass substrate. The conducting indium tin oxide is used as a gate and 275 nm of tantalum oxide (Ta_2_O_5_) as gate insulator over which 40 nm of zinc oxide (ZnO) as channel is deposited via radiofrequency sputtering.^20^ Aluminum is used as the top contact for the source and drain. The electrical characteristics are measured using Keysight B2902A. The device mechanism of the SE-FET is governed by a distinct redox reaction occurring within the insulator.^20^ When a positive gate voltage (*V*_GS_) is applied, oxygen ions and vacancies separate at the opposite interfaces of Ta_2_O_5_, leading to an additional electrolytic capacitance, as shown in Figure 2(a). During the reverse sweep of *V*_GS_, the rapid collapse of the internal electric field drives the capacitance negative,^23^ enabling steep switching without relying on filamentary effects. This electrolytic capacitance directly impacts memory decay by influencing charge retention. The redistribution of oxygen ions and vacancies over time leads to gradual changes in the internal field, resulting in a collapse of the internal field and a change in the resistance state of the channel. The gradual relaxation dynamics enable short-term memory, allowing the SE-FET to efficiently map past inputs into a high-dimensional state space and enhancing temporal pattern recognition, which can be leveraged for physical RC. Figure 2(b) shows the measured transfer and gate current characteristics of the SE-FET. The short-term memory of the SE-FET is depicted in Figure 2(c). Initially, the device is programmed using five write pulses at *V*_GS_ = 4 V with a frequency of 0.4 Hz. The response *I*_DS_ is then measured using read pulses *V*_DS_ = −1.5 V. In the absence of write pulses, the device current gradually returns to its initial state, as indicated by the blue arrow. Furthermore, unlike two-terminal devices, the drain terminal of the SE-FET can be used to control its short-term memory, as shown in Figure 2(d), where distinct decay constants are achieved by simply varying the read voltage at the drain terminal. This is because, in the off state, a positive *V*_Read_ increases the *V*_DS_ (drain–source potential) compared to the *V*_GS_, resulting in faster decay. In contrast, a negative *V*_Read_ makes *V*_GS_ relatively higher than *V*_DS_, increasing the conductance and decay time.

The device-to-device variation across three different SE-FET devices subjected to the same input of *V*_GS_ = 4 V at 1 Hz follows a consistent trend, differing only in the current magnitude, as shown in Figure 2(e). Additionally, when the same input is processed by the SE-FET at varying frequencies, it produces distinct output responses, as illustrated in Figure 2(f). This demonstrates the SE-FET’s ability to adapt to the different temporal dynamics of the input data.

### 2.2 Methods for Temporal Data Classification

To evaluate our SE-FET-based RC system using the MNIST data set, we digitize and crop the image sample from 28 × 28 to 24 × 24 by removing unused pixels. We use 60 000 images and a separate 10 000 sample set for training and testing, respectively. The readout network is implemented by using feedforward neural networks (FNNs) with one hidden layer consisting of 75 neurons. Training and testing are performed in Python using the Keras library.^24^ The neurons in the input layer are identical to the reservoir output per image, whereas 10 output neurons are labeled corresponding to digits “0” to “9”. Note that without a reservoir, an FNN requires 576 input neurons to represent all 24 × 24 pixels. However, with a reservoir, depending upon the length of the sequence (*L*) (often referred to also as a mask), the total number of reservoir outputs is 576/*L*. This is because only a single read operation is performed at the end of each sequence. A rectified linear unit (ReLU) is used as an activation function defined as *f*(*x*) = max(0, *x*) in the hidden layer. Where *x* is the input value and for positive input values, ReLU returns the input value itself, whereas for negative input values, ReLU outputs zero. For the output layer, the SoftMax activation function is used, defined as
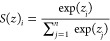
1where *z* is the input vector, ∑_*j*=1_^*n*^ exp(*z*_*j*_) is a normalization term to ensure that the value of the output vector *S*(*z*)*_i_* sums to 1, and *n* is the total number of output classes. The performance of the readout network is evaluated by the loss function categorical cross-entropy (CE)^25^ defined by
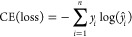
2where *y*_*i*_ is the true label for class *i* (1 for the correct class, 0 otherwise),  is the predicted probability for class *i*, and *n* is total number of output classes. The weight and bias in the readout network are updated during training using the Adam (Adaptive Moment Estimation) optimizer.

### 2.3 Methods for Time-Series Forecasting

A common way to test the performance of time-series forecasting in a physical reservoir is to use a chaotic system, such as the Mackey–Glass time series^26,27^ with a positive Lyapunov exponent.^28^ Such a system is sensitive to initial conditions such that a small error in the starting state can lead to significant differences in future behavior. A nonlinear time-delayed differential equation forms the basis of the Mackey–Glass time series.
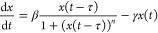
3This equation can display various kinds of behavior depending on the value of τ. Chaotic behavior occurs when τ is greater than 16.8. In this work, the goal of the task is to predict the next few steps (*t* + 1, *t* + 2, ..., *t* + *p*) of the time series by using the previous few times steps (*t*,*t* – 1, ..., *t* – *x*) of the response of the reservoir, where *t* is the present time step, *p* is the length of the predicted time step, and *x* is the length of the previous time step. The data set for this prediction task is obtained by solving the Mackey–Glass equation using the fourth-order Runge–Kutta for 1500 time steps. To obtain chaotic behavior, the parameters are set to β = 0.2, γ = 0.1, *n* = 10, and τ = 18. The time-series data obtained are then normalized between [0, 1] to reduce training time and improve the accuracy of prediction. The normalized Mackey–Glass time series is then fed as input, with a time step of 100 ms into a reservoir consisting of the SE-FET, and its temporal response recorded. The recorded response is then used to train the readout network with linear regression using scikit-learn library,^29^ which directly maps the recorded response to outputs using a simple weighted sum. During training and testing, the first 700 time steps are used for training, and the remaining 800 time steps are used for short-term prediction (*p* = 1); for long-term prediction *p* is set to 50. The performance of the readout network is evaluated by the loss function root mean squared error (RMSE) defined by
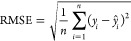
4where *y*_*i*_ is the actual values,  is the predicted values, and *n* is the total number of observations.

## 3 Results and Discussion

### 3.1 Temporal Data Classification

**Figure 3:**
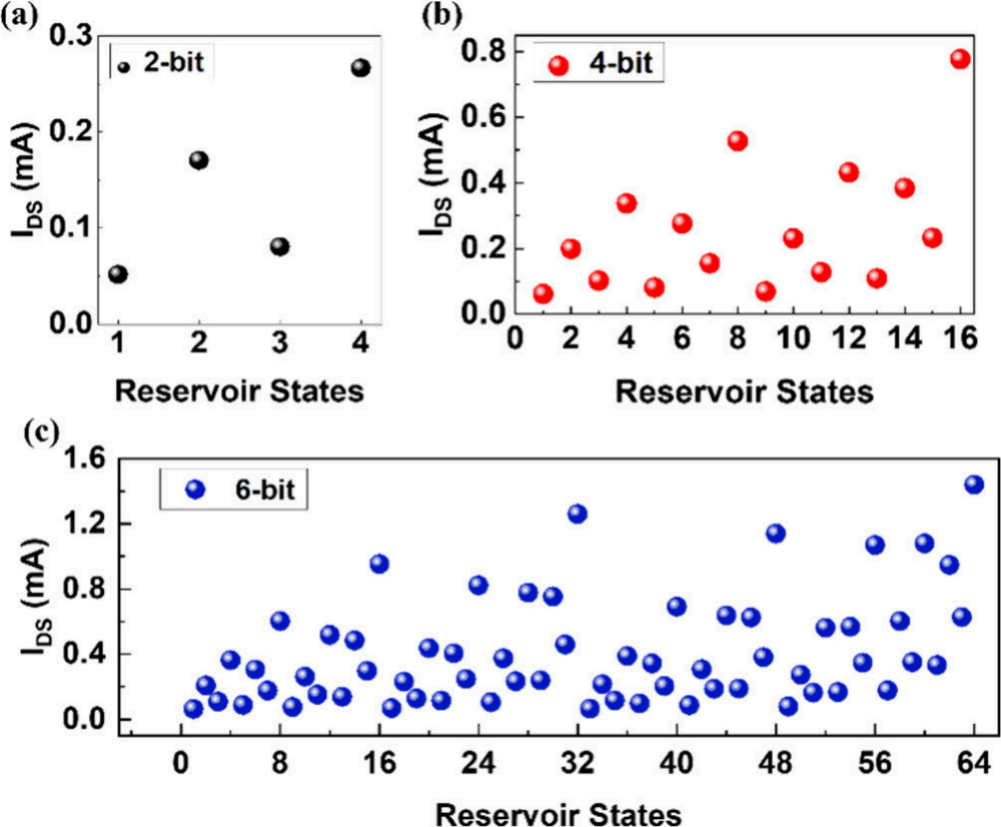
Reservoir states (*I*_DS_) show uniqueness in the temporal response of the SE-FET for binary sequences: (a) 2-bit (4 reservoir states) ‘00’- ‘11’; (b) 4-bit (16 reservoir states) ‘0000’–‘1111’; (c) 6-bit (64 reservoir states) for ‘000000’– ‘111111’.

Figure 3 shows the response of the SE-FET (i.e., the reservoir states) measured at the end of each sequence for different lengths of 2-bit, 4-bit, and 6-bit. To carry out these tests, each binary sequence is converted into a pulse stream, where each pulse with a period of 2.5 s, with 60% duty cycle, represents an input “1” @ 3 V and “0” @ 0 V, measured at *V*_Read_ = −1.5 V. To achieve a complete temporal response, the pulse stream duration should align with the memory decay time of the device. This is because pulses within this decay range influence the device state, whereas earlier pulses outside this range do not, as the device returns to its initial state.

The decay time of the SE-FET depends on factors such as the magnitude of *V*_Read_ and the applied input frequency. Different *V*_Read_ values lead to varying memory decay times (see Figure 2d). Additionally, the input frequency influences the magnitude of *I*_DS_, where a higher input frequency results in a lower *I*_DS_ value (see Figure 2f), causing the device to decay more rapidly. For a given experimental setup, the duration of the input sequence is determined by both *V*_Read_ and the applied input frequency.

**Figure 4:**
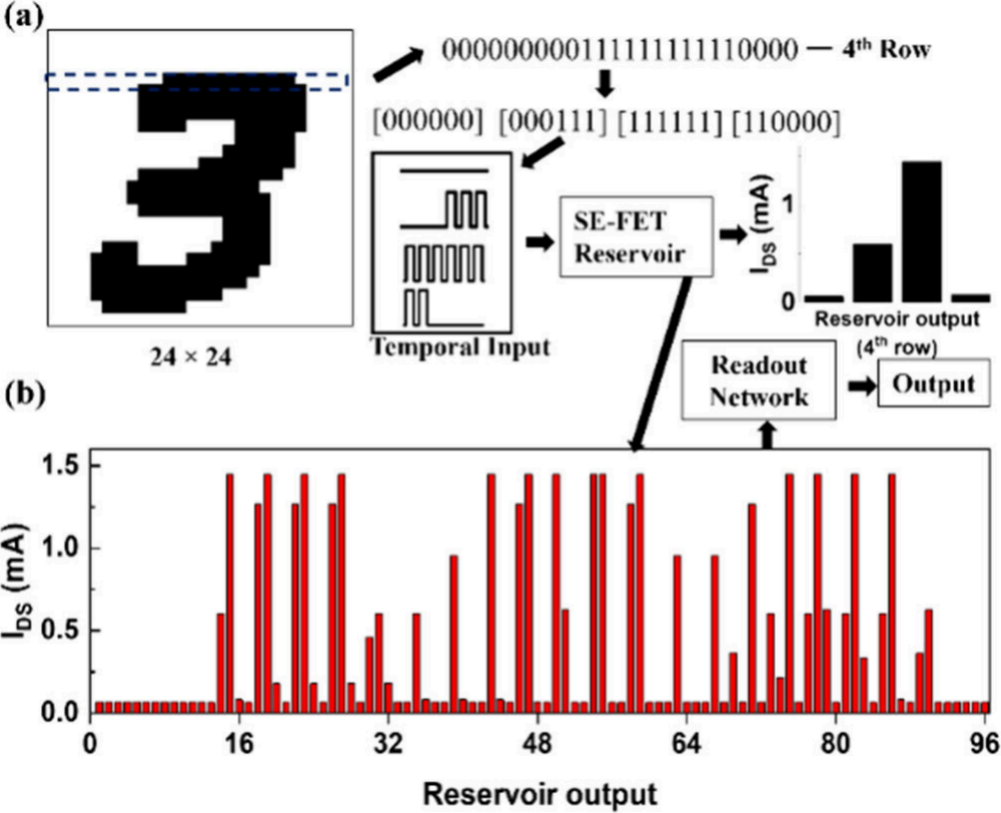
Process flow of the SE-FET-based reservoir system for classification of handwritten digits. (a) The reservoir response to a temporal input for row 4 of the image is exemplified. (b) The recorded reservoir output for digit 3 with a sequence length of 6-bits.

**Figure 5:**
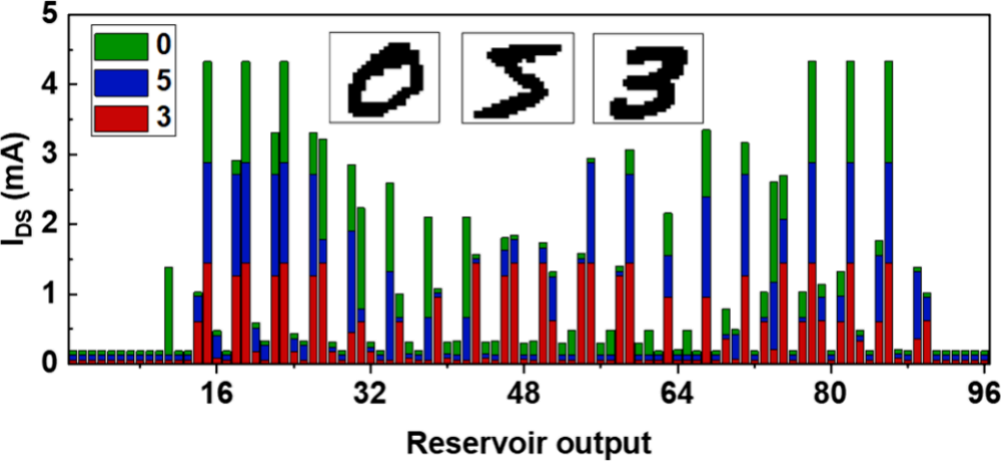
Stacked column plot showing reservoir output corresponding to the three examples (inset image of digits 0, 5, and 3) with sequence lengths of 6-bits. A significant difference in the reservoir output across three examples can be observed.

With the above considerations, it becomes necessary to have a memory time decay of at least 15 (2.5 × 6) s for a 6-bit sequence; therefore, a *V*_Read_ of −1.5 V is motivated by the need to achieve a longer memory decay time. The process flow of our SE-FET-based reservoir system for classification of handwritten digits is shown in Figure 4.The preprocessed image is first converted into a voltage sequential input by breaking down an entire row (containing 24 pixels) of an image into 24/L subsections. This is carried out in order to enhance the reservoir states, as an increment in *L* leads to exponential growth in reservoir states (2^*L*^), which may lead to redundancy. Furthermore, *L* is also restricted by the memory decay time of the device, as discussed above. With these considerations, each row of the image is fed into the SE-FET reservoir (as shown in Figure 4(a) for row number 4 with *L* = 6), and its output response (reservoir states) is recorded once at the end of each sequence. This is similarly repeated for all rows. As an example, all 96 (576/6) reservoir outputs for digit 3 are shown in Figure 4(b). These are fed to a trained readout network, for which an overall classification accuracy of 95.4% is obtained. Note that the reservoir output acts as an input to the readout network. Further, Figure 5 shows a stacked column plot that illustrates the reservoir output corresponding to three distinct examples.

**Figure 6:**
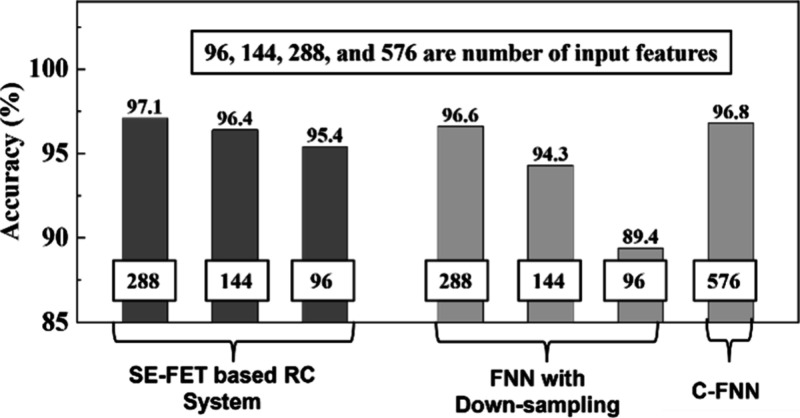
Comparison of the classification accuracy of three systems: the SE-FET-based RC system, a feedforward neural network (FNN) with down-sampling, and a conventional FNN (C-FNN) without any down-sampling, using the MNIST data set. The original input consists of 576 features (from a 24 × 24 pixel image). Sequence-based feature reduction reduces the input features for the readout network to 96, 144, 288, and 576, corresponding to sequence lengths (*L*) of 6, 4, 2, and 1, respectively, as features are read only once at the end of the sequence.

A significant difference in reservoir output for each example is observed, which contributes to the improved classification of digits by the readout network. We first benchmark the performance of our readout network with a reservoir against that of an equivalent model of a readout network without any reservoir, as shown in Figure 6. Here, the total number of input features is 576 (24 × 24); that is, pixels of the image are down-sampled (e.g., in a ratio of 6 to 1 for *L* = 6). Without any reservoir, an overall accuracy of 89.4% is achieved. An SE-FET-based reservoir outperforms a readout network without any reservoir by 6%, because its fading memory preserves temporal information. Even for smaller *L* = 2 and 4 our RC system shows performance improvement of 0.5% and 2.1%, respectively. Further, for *L* = 2 our SE-FET-based reservoir performs on par with normal FNNs (576 input features) without any down-sampling. In this case, our SE-FET-based reservoir achieves similar accuracy by using 50% less input features compared to a conventional FNN (C-FNN), thus reducing the training costs significantly without sacrificing accuracy of classification.

However, compared to *L* = 6, using *L* = 2 and *L* = 4 uses 200% and 50% more input features per image, respectively, for a slight improvement in accuracy of 1.7% and 1%, respectively. Next, the performance of our SE-FET reservoir is benchmarked against previously reported work in Table 1.

**Table 1:** Comparison of the Performance of the Solid Electrolyte FET-Based Reservoir with Reported Works for Image Classification

			**Readout network size**					
**Description [ref]**	**Reservoir output per image**	**Accuracy (MNIST)**	**Input**	**Hidden**	**Output**	**Sequence length**	**Write pulse for bit 1**	**Trainable parameters**
WO_*x*_memristor^12^	88	85.60%	88	-	10	5 bits	1.5 V, 0.5 ms	890
	176	88.10%	176	-	10		1.5 V, 0.8 ms	1770
SiO2: Ag memristor^13^	220	83%	220	-	10	4 bits	1.25 V, 50 μs	2210
ZnO/Ta_2_O_5_SE-FET^14^	576	91.19%	576	-	10	4 bits	3 V, 1.5 s	5770
SnO TFT^22^	196	90.73%	196	-	10	4 bits	5 V, 0.1 ms	1970
HfO_2_memristor^17^	196	90%	196	-	10	4 bits	4 V, 100 μs	1970
		95.10%	196	38	10			7876
Si_3_N_4_Leaky Fin-FET^18^	196	96%	196	128 × 64	10	4 bits	–5 V, 10 μs	34 122
ZnO/Ta_2_O_5_SE-FET [This work]	96	95.41%	96	75	10	6 bits	3 V, 1.5 s	8035

In refs (12)–^14^ and (22) readout networks consisting of only input and output layers are trained using logistic regression. Although this reduces the network size (meaning fewer parameters to train, (*input* + 1) × *output*), this results in a significant reduction in training costs. However, this is achieved at the expense of accuracy (83–91%). On the contrary, a reservoir with a readout network consisting of one or two hidden layers exhibits a much higher accuracy (95–96%).^17,18^ This is because hidden layers enhance the model’s ability to capture and leverage the intricate space–time correlations within the reservoir output. They enable the network to learn complex interactions between reservoir states, which a simple linear classifier would otherwise fail to recognize. This becomes especially crucial for classifying larger data sets like MNIST, where the reservoir provides richer feature extraction, and the hidden layers further refine these representations, significantly improving accuracy and robustness.

For a 4-bit sequence length in previous work, accuracies of 95.1% with a HfO_2_ memristor^17^ and 96% for a leaky Fin-FET^18^ were reported using reservoir outputs of 196 per image. In this work, by using a longer time frame reservoir with *L* = 6, a similar accuracy of 95.41% is achieved, but with an exceptionally small number of reservoir outputs per image (96). This reduction in reservoir outputs per image leads to a 51% decrease in reservoir size (number of devices) compared to refs (17) and (18), thereby reducing costs of hardware. Ideally, the total number of devices required in each reservoir is determined by the number of reservoir outputs per image, ensuring optimal alignment for parallel processing. This setup maximizes the efficient use of available devices, while maintaining a fixed connection between each input sequence and its corresponding device. Furthermore, the size of the readout network of our RC system is comparable to that in ref (17) and significantly smaller than that in ref (18), as shown in Table 1, which leads to a substantial reduction of training costs compared to ref (18).

### 3.2 Time-Series Forecasting

**Figure 7:**
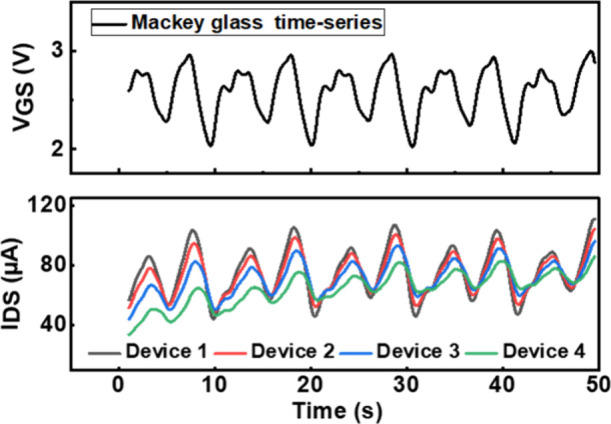
Temporal response of the SE-FET to the Mackey–Glass time series across four different devices.

**Figure 8:**
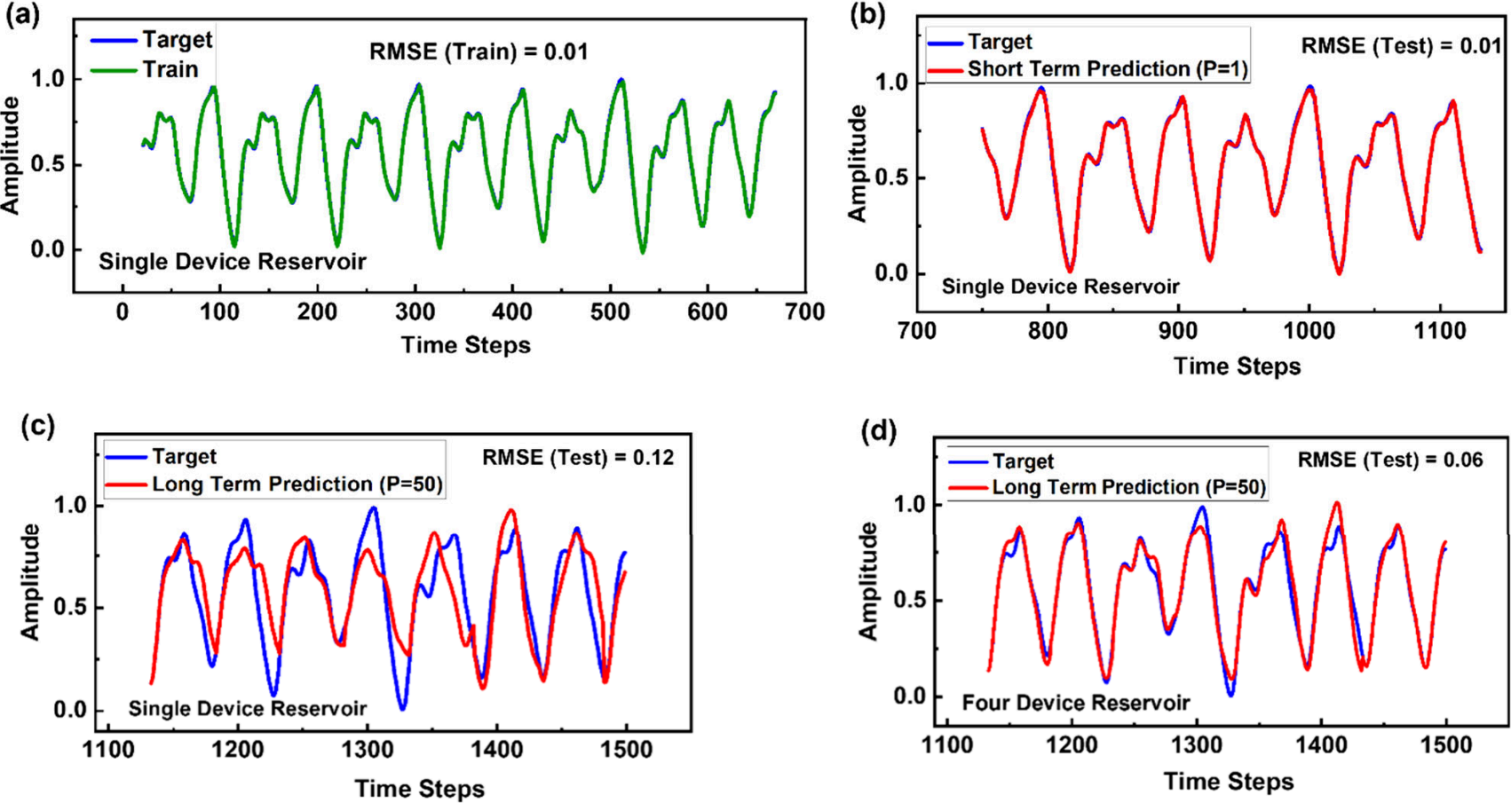
Training and testing performance of the readout network for the prediction of the Mackey–Glass time series. (a) Training performance of the readout network with a reservoir consisting of a single device. (b) Short-term prediction performance of the trained readout network using a single-device reservoir. (c) Long-term prediction performance using a single-device reservoir, resulting in an RMSE of 0.12. (d) Long-term prediction performance using a four-device reservoir, where four devices operate in parallel to process the same input. Their combined responses are concatenated to train the readout network, achieving an RMSE of 0.06, which represents a 50% improvement over that of the single-device reservoir.

The temporal response of the SE-FET across four different devices demonstrates device-to-device variability while effectively capturing the dynamic behavior of the Mackey–Glass time series as shown in Figure 7. In our first approach, we use a single SE-FET as a reservoir to implement the task of time-series forecasting. An excellent agreement is achieved between the target and the trained data, as shown in Figure 8(a). The network is then used to make short-term (*p* = 1) and long-term predictions (*p* = 50) of the time series as shown in Figure 8(b) and Figure 8(c) respectively.

The short-term prediction results in a low RMSE = 0.01, whereas the long-term prediction results in a slightly higher RMSE = 0.12. This can be further improved using the responses from four devices instead of just one. In this approach, four different devices operate in parallel to process the same input, and their combined responses are concatenated to train the readout network. For *p* = 50, a low error rate of RMSE = 0.06 is achieved, which is a 50% improvement from the single device-based reservoir, as shown in Figure 8(d).

For the same input, different SE-FET devices follow similar trends but vary in amplitude (*I*_*DS*_) as shown in Figure 7. These naturally occurring variations in device characteristics enrich the reservoir’s computational diversity, leading to enhanced performance particularly in tasks like chaotic time-series forecasting. Instead of viewing variability as purely detrimental, it can be strategically leveraged to improve adaptability and generalization. Optimizing variations in fabrication, such as tuning material properties, deposition uniformity, and device heterogeneity, could further enhance the effectiveness of the physical RC by balancing randomness and stability for improved predictive accuracy.

**Figure 9:**
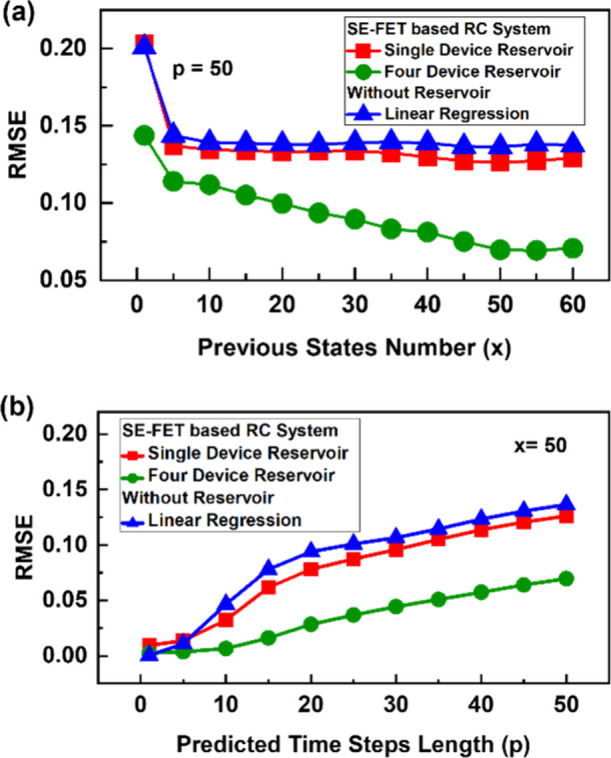
A comparison of performance of an SE-FET-based reservoir with a conventional machine learning algorithm (linear regression) for time-series forecasting. (a) The performance comparison for long-term prediction *p* = 50 as a function of number of previous states (*x*). (b) A comparison of the performance for increasing predicted length (*p*), keeping the number of previous states fixed at *x* = 50.

**Figure 10:**
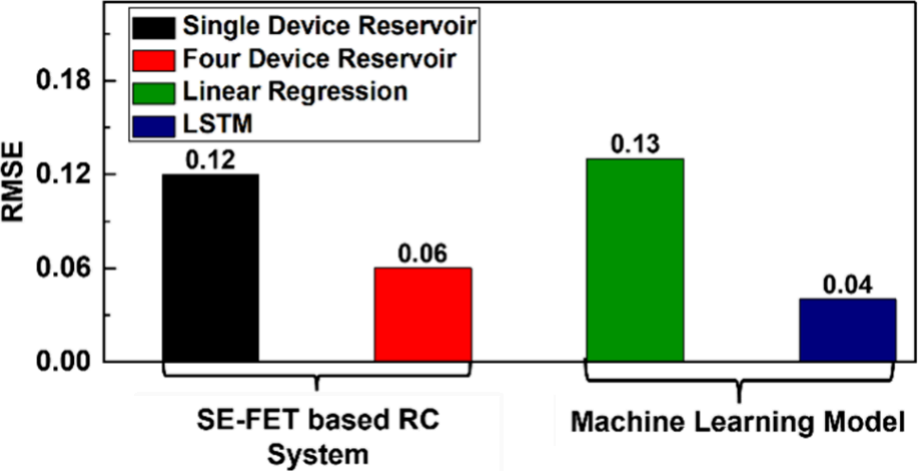
Comparison of long-term time-series forecasting performance (*p* = 50) between the SE-FET-based RC system and state-of-the-art machine learning models such as linear regression and long short-term memory (LSTM) on the chaotic Mackey–Glass data set.

The performance of our SE-FET-based reservoir is first benchmarked with a conventional network (without any reservoir), both trained using the same algorithm (linear regression) for forecasting the Mackey–Glass time series, as shown in Figure 9(a,b). The SE-FET-based RC system outperforms a conventional machine learning algorithm for both single- and four-device-based reservoirs. In Figure 9(a) we compare the performance of our RC system to that of a conventional network for long-term prediction with *p* = 50, where we vary the number of previous states (*x*) to study its impact on system performance. We train and test all three systems with variable *x* and plot their performance in Figure 9(a). These results show that the performance of the conventional and single-device-based reservoir saturates after 10 previous states. On the other hand, in a four-device reservoir, gradual improvement up to 50 previous states is observed with RMSE = 0.06. We also study the impact of the predicted length (*p*), keeping the number of previous states fixed at *x* = 50. With an increasing number of predicted lengths, it becomes much more challenging due to the chaotic nature of the Mackey–Glass time series. Here also our four-device-based reservoir outperforms conventional and single-device-based reservoirs, showing lower deterioration in performance with increasing predicted length, as shown in Figure 9(b). These results reveal that using more SE-FET devices to increase the reservoir size may lower prediction errors and extend the duration of accurate prediction. Additionally, we compared our SE-FET-based RC system with a long short-term memory (LSTM) model for long-term time-series forecasting. The LSTM, implemented in Keras,^30^ featured a single 45-unit LSTM layer and a dense output layer with 50 neurons, matching our four-device SE-FET reservoir in a setup for prediction with (10 760 vs 10 050) trainable parameters for the LSTM and RC, respectively. As shown in Figure 10, the LSTM achieved a lower RMSE (0.04) than our SE-FET RC system (0.06). This is consistent with literature highlighting the superiority of state-of-the-art machine learning models such as LSTM in capturing long-term dependencies and complex temporal patterns over reservoir computing.^9,31^

However, despite having a similar number of trainable parameters, LSTMs are significantly more computationally expensive due to their recurrent connections and four internal weight matrices (input, forget, cell, and output gates), leading to increasing complexity per time step. Backpropagation through time further adds to memory overhead due to the requirement to store intermediate states. In contrast, our SE-FET-based RC system captures temporal dependencies through computation-in-memory processing, eliminating recurrent computations. With a simpler linear regression readout, it reduces memory and computational costs while maintaining competitive performance, making it ideal for resource-constrained, low-latency edge computing applications.

Next, we benchmark the performance of the SE-FET-based reservoir with previously reported work, as shown in Table 2. In this work, we demonstrate an SE-FET-based RC system that uses 50 previous states to predict the next 50 states without any feedback loop (i.e., no predicted data fed back into the reservoir as new input) or update cycle in contrast to previously reported work.^9,22^ This makes the implementation of this approach efficient and straightforward. Further, our reservoir consists of four devices, with each device considering 50 previous states, resulting in a total of 200 (4 × 50) input features to the readout network. This is a significant reduction compared to a WO*x* memristor-based reservoir,^9^ which uses 1000 features for predicting a single time step.

**Table 2:** Summary of Various Physical Reservoir Computing Implementations for the Task of Time-Series Forecasting

**Description [ref]**	**Number of devices**	**Prediction length**	**Time-series data**	**Error**
WO_*x*_ memristor^9^	20	50	Mackey–Glass	∼0.2 (MASE)
TiO*x*/TaO*y* memristor^15^	25	1	Henon map	0.04 (NRMSE)
SnO TFT^22^	3	50	Mackey–Glass	0.08 (NRMSE)
ZnO/Ta_2_O_5_SE-FET [this work]	4	50	Mackey–Glass	0.06 (RMSE)

Our longer time scales, though slower than conventional two-terminal devices, offer advantages in energy-efficient, event-driven AI systems, making them well-suited for applications such as smart sensors, IoT, and wearable devices. In these scenarios, slower dynamics align naturally with input signals, enabling efficient processing. However, if a faster operation is required, the device speed can be significantly enhanced through scaling and optimization.

Beyond MNIST classification and Mackey–Glass series forecasting, scaling to larger data sets or more complex tasks such as high-resolution color image classification, which involves multichannel (RGB) dependencies and intricate spatial relationships, or large-scale speech processing, which requires fine-grained temporal pattern extraction over long durations, will demand handling higher input dimensionality, complex feature interactions, and high computational resources. Extending the system to these applications may benefit from hierarchical reservoirs,^32^ where multiple layers progressively extract more abstract features, and adaptive learning mechanisms, which dynamically tune reservoir properties (e.g., conductance, short-term memory) to enhance scalability and performance.

## 4 Conclusions

We experimentally demonstrate a read voltage controlled decay time of the SE-FET, which enables an extended temporal input of 6 bits to a reservoir, with a classification accuracy of 95.41%, while utilizing a notably lowest number of reservoir outputs (96) per image. Consequently, this reduces the hardware and training costs of edge systems significantly. We demonstrate a simple enhanced framework for long-term chaotic time-series forecasting without feedback and with a reduced number of devices. Further, the use of a separate control terminal for read and write operation simplifies the reservoir implementation compared with that in two-terminal devices.
